# Mono- and Intralink Filter (Mi-Filter) To Reduce False Identifications in Cross-Linking Mass Spectrometry Data

**DOI:** 10.1021/acs.analchem.2c00494

**Published:** 2022-12-12

**Authors:** Xingyu Chen, Carolin Sailer, Kai Michael Kammer, Julius Fürsch, Markus R. Eisele, Eri Sakata, Riccardo Pellarin, Florian Stengel

**Affiliations:** † Department of Biology, University of Konstanz, Universitätsstrasse 10, Konstanz 78457, Germany; ‡ Konstanz Research School Chemical Biology, University of Konstanz, Universitätsstrasse 10, Konstanz 78457, Germany; § Department of Molecular Structural Biology, Max Planck Institute of Biochemistry, Martinsried 82152, Germany; ∥ Institute for Auditory Neuroscience, University Medical Center Göttingen, Göttingen 37077, Germany; ⊥ Structural Bioinformatics Unit, Department of Structural Biology and Chemistry, Institut Pasteur, CNRS UMR 3528, 28 rue du Docteur Roux, Paris 75015, France

## Abstract

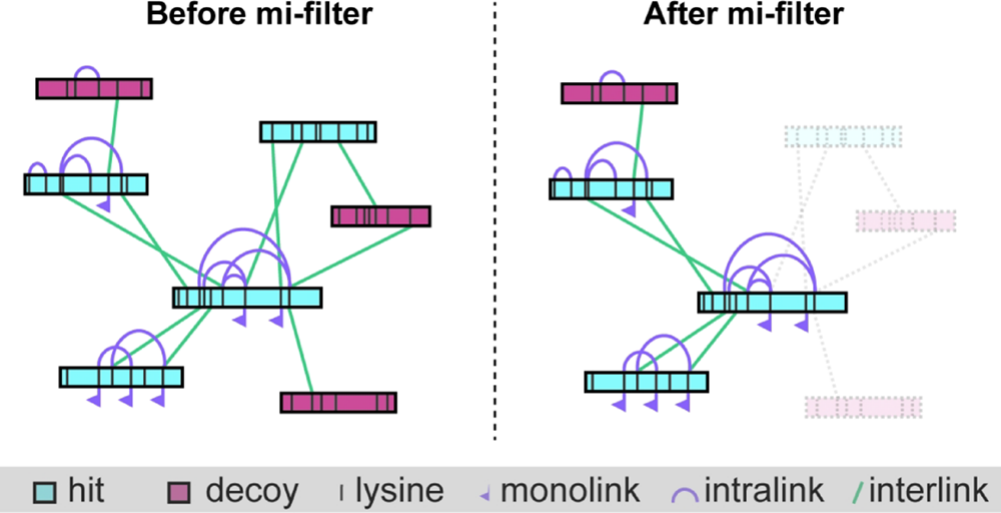

Cross-linking mass spectrometry (XL-MS) has become an indispensable tool for the emerging field of systems structural biology over the recent years. However, the confidence in individual protein–protein interactions (PPIs) depends on the correct assessment of individual inter-protein cross-links. In this article, we describe a mono- and intralink filter (mi-filter) that is applicable to any kind of cross-linking data and workflow. It stipulates that only proteins for which at least one monolink or intra-protein cross-link has been identified within a given data set are considered for an inter-protein cross-link and therefore participate in a PPI. We show that this simple and intuitive filter has a dramatic effect on different types of cross-linking data ranging from individual protein complexes over medium-complexity affinity enrichments to proteome-wide cell lysates and significantly reduces the number of false-positive identifications for inter-protein links in all these types of XL-MS data.

## Introduction

An increasingly relevant approach for addressing protein–protein interactions (PPIs) is based on the rapidly evolving technology of cross-linking coupled to mass spectrometry (XL-MS). The general approach of protein XL-MS is based on covalent bonds that are formed using cross-linking reagents between proximal functional groups (most commonly lysine residues) in their native environment.^1−4^ The actual cross-linking sites are subsequently identified by mass spectrometry (MS) and reflect the spatial proximity of regions and domains within a given protein (intra-link) or between different proteins (inter-link). Additionally, the cross-linker can react twice within one peptide (loop-link) or only on one side with the peptide and hydrolyze on the other side (mono-link), revealing information on the accessibility of a specific amino acid residue. The field has seen significant technological and conceptual progress over the last couple of years, and by now, various enrichment strategies, different cross-linking chemistries, and multiple detection and annotation strategies have been introduced.^1,2,4^

With the structural probing of recombinantly expressed static protein complexes now being firmly established, the recent applications of XL-MS on the systems level^5^ and in living cells^6^ that has spurred great interest and an ever-increasing number of studies ranging from bacterial, fungal, and mammalian cell lysates and cultured cells,^7,8^ specific cellular organelles,^9−11^ and tissues^12,13^ have been reported. These studies hint at the exciting prospect that XL-MS will soon be able to facilitate the structural probing of interaction partners of any protein of interest within living cells or even organisms.

However, the confidence in individual protein–protein interactions (PPIs) based on cross-linking data depends on the correct assessment of individual inter-protein cross-links. As recent data show that erroneous assignments in cross-linking data are frequently underestimated,^14^ which is particularly the case for inter-protein cross-links,^15^ this can undermine the confidence in individual PPIs and protein networks based on cross-linking data.

In this article, we describe a novel mono- and intralink filter (mi-filter) that is applicable to any kind of cross-linking data and analysis pipelines. It stipulates that only proteins for which at least one monolink or intra-protein cross-link has been identified within a given data set should be considered for an inter-protein cross-link and therefore participate in a PPI. It is based on the observation that if the abundance of protein is high enough to be detectable by XL-MS, the formation rate of monolinks and intra-protein cross-links will be significantly higher than that of interlinks.^16^ In other words, if no mono-link or intra-protein cross-link can be detected for a given protein, there is a high likelihood that this protein is not addressable by XL-MS in this particular sample and any inter-protein cross-link that includes this protein is likely a false PPI.

We show that this simple and intuitive filter has a dramatic effect on all types of cross-linking data ranging from single protein complexes, over medium-complexity affinity enrichments to proteome-wide settings, and significantly reduces the number of false-positive identifications in all these types of XL-MS data.

## Experimental Section

### Mi-Filter Script

The mi-filter script was written in python and is available at the Github repository (https://github.com/stengellab/mi-filter.git). It is tailored to xQuest^17^ output tables but can, in principle, be applied to cross-linking MS data sets obtained by any of the established cross-link-identification software platforms such as MeroX,^18^ Xlinkx,^19^ Xi,^20^ pLink2,^21,22^ or RNPxl.^23^ It selects proteins from the input data set, which contain at least one mono- or intra-protein link and subsequently filters for inter-protein cross-links within this list. It also calculates a decoy ratio (using the ratio of the target and decoy links) at each ld-Score cutoff for monolinks and inter-protein and intra-protein cross-links separately.

In detail, the mi-filter script works as follows: it filters the input files for a specified ld-Score, then concatenates the input data frames, and, if specified, filters for biological replicates of cross-linking sites (therefore, input files must be sorted by biological replicates). In the next step, it adds a “decoy” column to the concatenated data frame. In the “XLtype” column, strings are replaced in a way that only three types of cross-link are left: monolinks and intra-protein and inter-protein cross-links. Proteins without a monolink or an intra-protein cross-link are then filtered out by the parameter “–mi” when running the mi-filter program.

### 26S Proteasome Cross-Linking Data Set

Purification of yeast 26S proteasomes was performed as described in a previous study.^24^*S. cerevisiae* cells (YYS40; MATa rpn11::RPN113FLAG-HIS3) were grown for 48 h and harvested in the stationary phase. The purification of 3XFLAG-tagged 26S proteasome was carried out by affinity purification using M2 anti-FLAG beads (Sigma A2220). After incubation for 1.5 h at 4 °C, the proteasome was eluted with FLAG peptide. An overnight sucrose gradient was carried out for the second purification step. The sucrose gradient was centrifuged in a Beckman SW41 rotor for 17 h at 4 °C at 28,000 rpm. Proteasome-containing fractions were identified by the degradation of the peptide suc-LLVY-AMC, SDS-PAGE analysis, and Bradford assay. Purified 26S proteasome (1 μg/μL) (100 μg) were subsequently incubated with the isotopically labeled cross-linking reagent disuccinimidyl suberate d0/d12 (DSS-H12/D12, Creativemolecules Inc.) at a final concentration of 1 mM for 30 min at 30 °C while shaking at 650 rpm in a Thermomixer (Eppendorf). The reaction was quenched with ammonium bicarbonate at a final concentration of 50 mM for 10 min at 30 °C and 650 rpm. Cross-linked samples were dried (Eppendorf, Concentrator plus), resuspended in 100 μL of 8 M Urea, reduced, alkylated, and digested with trypsin (Promega). Digested peptides were separated from the solution and retained by a solid-phase extraction system (SepPak, Waters). Cross-linked peptides were enriched by size exclusion chromatography using an ÄKTAmicro chromatography system (GE Healthcare) equipped with a SuperdexTM Peptide 3.2/30 column (column volume = 2.4 mL). Fractions were collected in 100 μL units and analyzed by liquid chromatography-tandem mass spectrometry (LC–MS/MS). For each cross-linked sample, two fractions (1.2–1.3 mL and 1.3–1.4 mL) were collected and measured in technical duplicates. Absorption levels at 215 nm of each fraction were used to normalize peptide amounts prior to LC–MS/MS analysis.

LC–MS/MS analysis was carried out on an Orbitrap Fusion Tribrid mass spectrometer (Thermo Electron, San Jose, CA). Peptides were separated on an EASY-nLC 1200 system (Thermo Scientific) at a flow rate of 300 nL/min over an 80 min gradient (5% acetonitrile in 0.1% formic acid for 4 min, 5–35% acetonitrile in 0.1% formic acid in 75 min, and 35–80% acetonitrile in 1 min). Full scan mass spectra were acquired in the Orbitrap at a resolution of 120,000, a scan range of 400–1500 *m/z*, and a maximum injection time of 50 ms. Most intense precursor ions (intensity ≥5.0 × 10^3^) with charge states 3–8 and monoisotopic peak determination set to “peptide” were selected for MS/MS fragmentation by CID at 35% collision energy in a data-dependent mode. The duration for dynamic exclusion was set to 60 s. MS/MS spectra were analyzed in the iontrap at a rapid scan rate.

For the cross-link identification of the 26 proteasome in a “proteome-wide setting,” a database was compiled which contained the 34 proteins of the 26S proteasome plus the 200 most abundant proteins in *S. cerevisiae* as annotated in the PAX database (https://pax-db.org/). MS raw files were subsequently converted to centroid files and searched using xQuest in ion-tag mode. Cross-links were exported as .tsv files with the filter settings Δ*S* = 95 and a max. ppm range from −5 to 5, containing all (nonunique) identifications. The mi-filter was applied to different ld-Score cut-offs (20, 25, 28, and 32) before comparing the ratio of target to decoy hits for each data set before and after mi-filtering (Supplementary Data 1 and Figure 3A,B).

### Pre-60S Ribosome XL-MS Data Set

The data set consists of biological triplicate measurements of 12 different pre-60S ribosomal particles, which were enriched using affinity-tagged RBFs and were collected as part of another study.^25^ The mi-filter was applied to different ld-Score cut-offs (20, 25, 28, and 32) and target to decoy hits compared before and after mi-filtering (Supplementary Data 2 and Figure 3C,D).

### Proteome-Wide Cross-Linking Data Set

The data set contains biological triplicate measurements of cell lysate in *Saccharomyces cerevisiae* and was collected as part of another study.^16^ xQuest results from this paper were directly downloaded and the cross-linked sample using equimolar concentrations (1×) of BS3 as a cross-linker was chosen for further analysis. The mi-filter was applied to different ld-Score cut-offs (20, 25, 28, and 32) and target to decoy hits compared before and after mi-filtering (Supplementary Data 3 and Figure 3E,F).

### Mapping of Filtered Cross-Links

Cross-link networks were visualized with xiNET.^26^

### Data Availability

The MS raw files, the cross-link databases, and the original xQuest result files have all been deposited to the ProteomeXchange Consortium via the PRIDE partner repository^27^ with the project accession number PXD031215. The previously published ribosome^25^ and lysate^16^ data sets have the project accession numbers PXD021831 and PXD014759, respectively.

## Results

### Concept of the Mi-Filter

**Figure 1:**
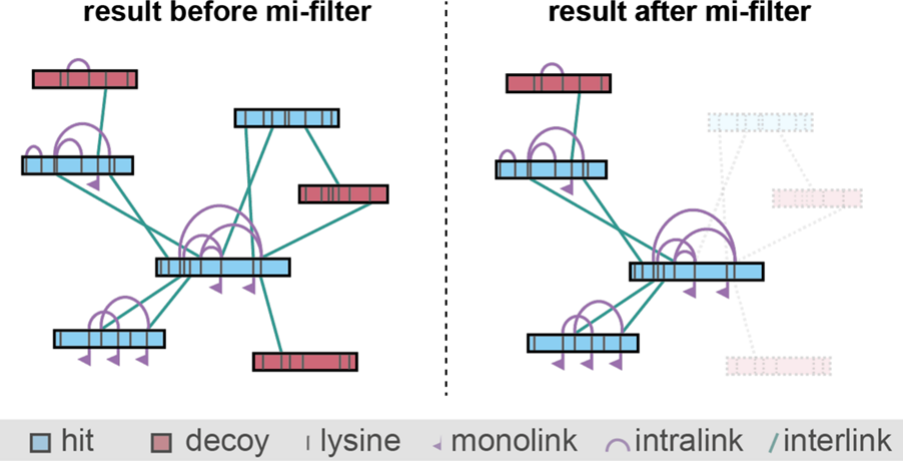
Concept of the mi-filter. Only proteins that contain at least one identified monolink or intra-protein cross-link are considered for inter-protein cross-links and can therefore be part of a PPI.

Our “mi-filter” (monolink/intralink filter) is based on the simple idea that only proteins for which at least one monolink or intra-protein cross-link has been identified within a given data set should participate in an inter-protein cross-link and be part of a legitimate PPI (Figure 1).

Our approach is not designed as a contradiction to standard FDR calculations^11,14,19,28−30^ but is rather intended as an additional tool that can be applied on the top of the existing workflows and before the final FDR estimation in order to minimize false-positive assignments of inter-protein cross-links.

### Inter-Protein Cross-Links Are Disproportionally Affected by False-Positive Assignments

**Figure 2:**
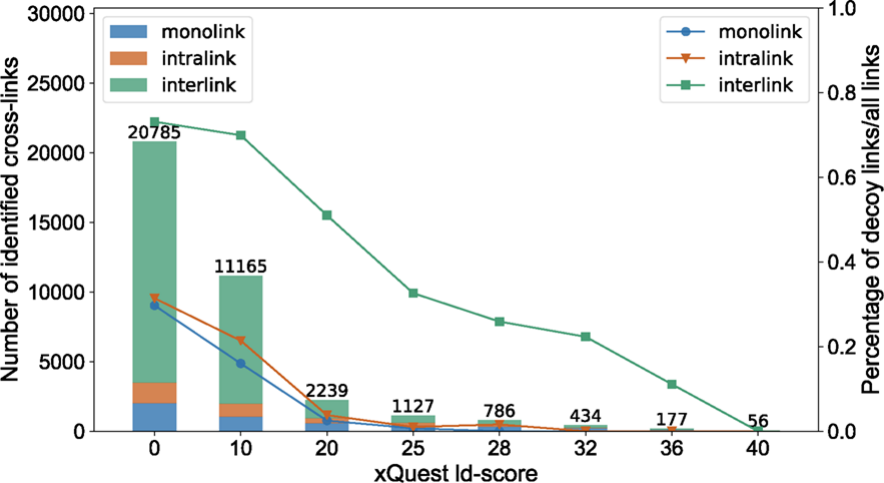
Inter-protein cross-links are disproportionally affected from false-positive assignments. Bar chart shows the cumulative amount of detected monolinks and intra-protein cross-links and inter-protein cross-links (*y*-axis, left) of the 26S proteasome at different ld-score cut-offs,^31^ that is, increasing levels of agreement between the measured experimental and in-silico-generated reference spectra. The relative proportion of detected decoy hits to all detected hits (*y*-axis, right) versus the respective ld-score setting (*x*-axis) is plotted on the right *y*-axis (symbols).

Minimizing false-positive assignments for inter-protein cross-links is particularly crucial as all PPIs based on cross-linking data depend entirely on information from inter-protein cross-links (Figure 2). Figure 2 shows the amount of detected hits for monolinks and intra-protein and inter-protein cross-links for the 26S proteasome at increasingly stringent filtering settings, that is, increasing agreement between measured experimental and in-silico-generated reference spectra. The decoy ratio is the relative proportion of detected decoy hits to all detected links. The data show that the relative proportion of detected decoy hits for inter-protein cross-links is significantly larger than that for mono or intra-protein links for all settings and, importantly, that inter-protein cross-links still contain a significant number of false-positive identifications at cut-offs where the number of detected decoys for intra-protein and monlinks are already negligible.

### Mi-Filter Reduces False-Positive Assignments for Inter-Protein Cross-Links for Different Types of Cross-Linking Data

**Figure 3:**
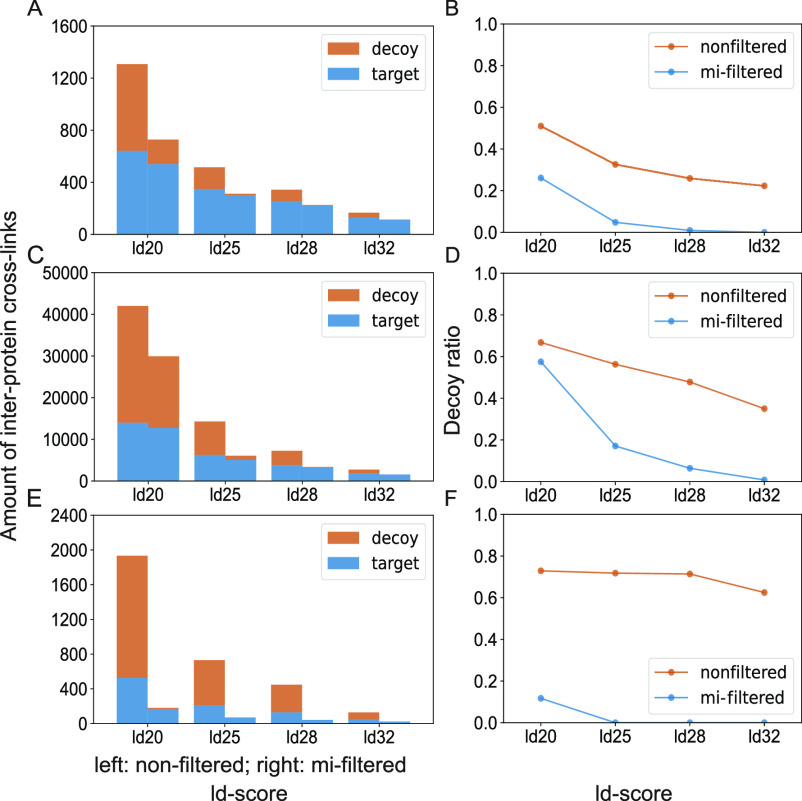
Comparison of false-positive assignments for inter-protein cross-links with and without the mi-filter. Inter-protein cross-links are shown for three different types of datasets representing typical experimental set-ups including the 26S proteasome from *S. cerevisiae* as an example of an individual protein complex (A and B), affinity enrichments of pre-60S ribosomal particles (C and D), and a proteome-wide cross-linking experiment using *S. cerevisiae* cell lysate (E and F). Panels A, C, and E show the number of decoy and target hits (nondecoy hits) in nonfiltered (left bar in each group) and mi-filtered samples (right bar in each group) for increasingly stringently filtered data. Target hits are shown in blue and decoys in red. Panels B, D, and F show the ratio of decoy to target hits of nonfiltered (red line) versus mi-filtered results (blue line) for the respective data sets.

In order to evaluate the effect of the mi-filter on false-positive assignments of inter-protein cross-links, we applied it to typical cross-linking data sets of different complexities (Figure 3).

Our least complex sample is the 26S proteasome from *S. cerevisiae* consisting of 34 proteins (Figure 3A,B). An intermediate one is the combined data set of pre-60S ribosomal particles obtained by affinity enrichment, containing a total of around 300 proteins (Figure 3C,D). We could previously show that the application of the mi-filter to this data set results in significantly reduced false-positive assignments,^25^ but only now, we have thoroughly investigated the influence of the mi-filter on this and other data sets and for various settings of increasingly stringent filtering. The most complex sample we evaluated using our mi-filter is a proteome-wide XL-MS data set of *S. cerevisiae* cell lysate^16^ (Figure 3E,F).

We first had a closer look at the relative abundance of proteins that were filtered out by the mi-filter, taking the pre-60S ribosomal particle data set as an example.^25^ Here, proteins for which a mono- or intra-protein link was detected are in average of significantly higher abundance than proteins without mono- or intra-protein links (Supplementary Figure 1). This effect has been noted also previously for other data sets^30^ and already indicates that proteins without mono- or intra-protein links are either not present in the sample at a concentration high enough for cross-link identification or they are not present at all.

After the application of the mi-filter (right bar of each group), all data sets consistently exhibit a significant decrease in the number of detected decoy inter-protein links (Figure 3A–F). It is interesting to note that this is not only true for the different sample types but also for the increasingly stringent filtering settings (i.e., increasingly good matches between experimental data and in-silico-generated reference spectra), where the mi-filter is able to filter out most decoy links already at medium filtering settings (Figure 3). We have then used the high-resolution structure of the *S. cerevisiae* 26S proteasome (PDB 4CR2) and a cutoff of 35 A° (the maximal lysine Cα–Cα distance that our cross-linker can bridge) to generate a control data set with structurally compatible cross-links (bona fide true-positive links) to assess the sensitivity of the mi-filtered data. Using this data set, the mi-filter also demonstrates very good sensitivity as it is able to retain the majority (>90%) of bona fide true-positive inter-protein cross-links (Supplementary Table 1).

Taken together, this demonstrates the value of the mi-filter as a stringent filtering device that results in a significant reduction of false inter-protein cross-link identifications in different types of cross-linking data.

### Structural Accuracy of the mi-Filtered Data

In the next step, we wanted to test and benchmark the mi-filter also for its ability to identify true-positive cross-links in a proteome-wide setting. In contrast to mixtures of purified proteins or protein complexes, which can be benchmarked against existing atomistic high-resolution structures in order to assess true-positive identifications, there is no known ground truth in a proteome-wide cross-linking experiment, as the precise protein arrangement within a cell or lysate is unknown.

**Figure 4:**
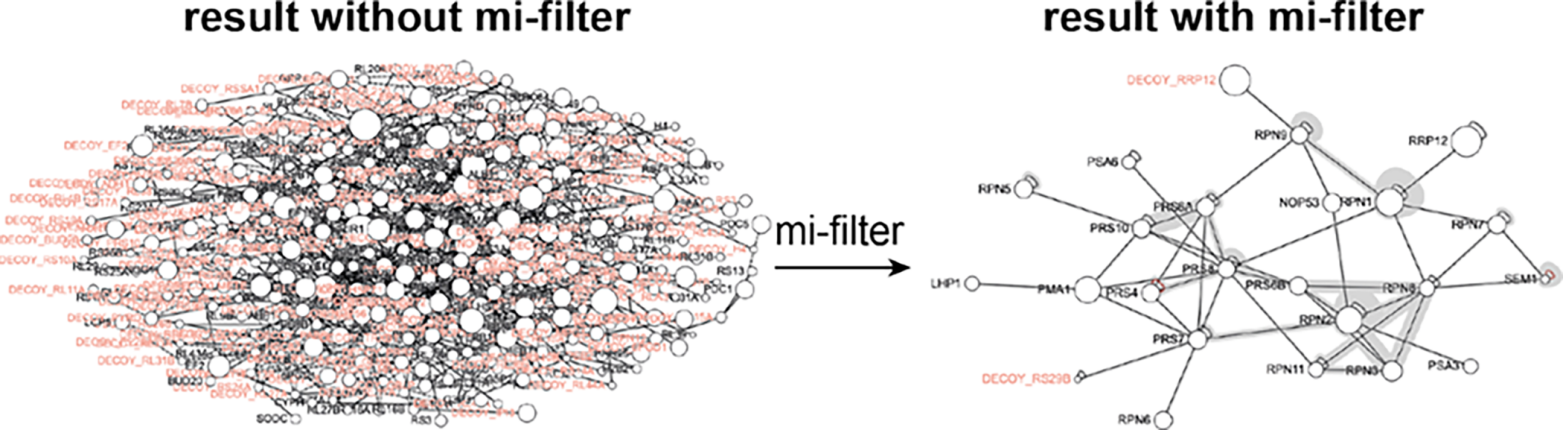
Quality of the mi-filtered data. Cross-linking data set of the 26S proteasome represented as a network graph where proteins are shown as white nodes and inter-protein links as gray lines. Graph is drawn at the constant score cutoff of Id-25 with and without the application of the mi-filter and the data were searched against a manually curated database mimicking proteome-wide protein distribution (see Experimental Section and Supplementary Figure 2 for details).

We, therefore, took the totality of MS and MS/MS spectra that we had experimentally obtained from a sample of cross-linked 26S proteasome and searched it in a proteome-wide setting (i.e., against a large protein database; see Experimental Section for details) using a constant score cutoff with and without the application of the mi-filter (Figure 4). Our data show that the application of the mi-filter did not only lead to a significant reduction of detected decoy hits. When mapped again onto the published high-resolution cryogenic electron microscopy structure of the *S. cerevisiae* 26S proteasome (PDB 4CR2), over 90% of our mi-filtered interprotein cross-links fall within 35 A° (Supplementary Figure 2), indicating that our mi-filtered cross-links are also structurally accurate.

## Discussion

In this manuscript, we describe and benchmark a mono- and intralink filter that is in principle applicable to any kind of cross-linking data and analysis pipelines. This simple and intuitive mi-filter, which removes inter-protein cross-links if the connected polypeptides are not additionally represented within their respective intralink or monolink pools, reduces false-positive identifications for inter-protein cross-links significantly. We show that this is true for different types of cross-linking data ranging from individual protein complexes, over medium-complexity affinity enrichments to proteome-wide settings. Moreover, in addition to reliably reducing the amount of detected decoy hits in a given cross-linking sample, the mi-filter is also able to identify and retain the majority of true-positive cross-links, suggesting very good sensitivity.

While we have used the *xQuest* cross-linking software in this manuscript as an example to identify cross-links, our mi-filter can in principle be applied to any cross-linking software. We therefore suggest its use as a tool to minimize false-positive assignments for inter-protein links prior to FDR estimation using the respective workflow of choice.

Taken together, our mi-filter greatly enhances the reliability of individual inter-protein cross-links in any type of cross-linking data and therefore their ability to provide reliable and biologically relevant positional information as a source of a PPI.

## Supporting Information Available

Relative abundance of inter-protein cross-links with and without additional mono- or intra-protein cross-link; mapped distances of all inter-protein cross-links within the mi-filtered 26S proteasome data set at Id 25; and mapping of identified cross-links with and without mi-filtering (PDF)
Protein cross-links of the 26S proteasome, mi-filtered (large database) (XLSX)
Protein cross-links of 60 S ribosome biogenesis intermediates, mi-filtered (XLSX)
Protein cross-links of lysate, mi-filtered (XLSX)

## References

[ref1] Piotrowski C.; Sinz A. Structural Investigation of Proteins and Protein Complexes by Chemical Cross-Linking/Mass Spectrometry. Adv. Exp. Med. Biol. 2018, 1105, 101–121. 10.1007/978-981-13-2200-6_8. 30617826

[ref2] Yu C.; Huang L. Cross-Linking Mass Spectrometry: An Emerging Technology for Interactomics and Structural Biology. Anal. Chem. 2018, 90, 144–165. 10.1021/acs.analchem.7b04431. 29160693 PMC6022837

[ref3] Leitner A.; Faini M.; Stengel F.; Aebersold R. Crosslinking and Mass Spectrometry: An Integrated Technology to Understand the Structure and Function of Molecular Machines. Trends Biochem. Sci. 2016, 41, 20–32. 10.1016/j.tibs.2015.10.008. 26654279

[ref4] O’Reilly F. J.; Rappsilber J. Cross-linking mass spectrometry: methods and applications in structural, molecular and systems biology. Nat. Struct. Mol. Biol. 2018, 25, 1000–1008. 10.1038/s41594-018-0147-0. 30374081

[ref5] Chavez J. D.; Bruce J. E. Chemical cross-linking with mass spectrometry: a tool for systems structural biology. Curr. Opin. Chem. Biol. 2019, 48, 8–18. 10.1016/j.cbpa.2018.08.006. 30172868 PMC6382564

[ref6] Matzinger M.; Mechtler K. Cleavable Cross-Linkers and Mass Spectrometry for the Ultimate Task of Profiling Protein-Protein Interaction Networks in Vivo. J. Proteome Res. 2021, 20, 78–93. 10.1021/acs.jproteome.0c00583. 33151691 PMC7786381

[ref7] Klykov O.; Steigenberger B.; Pektas S.; Fasci D.; Heck A. J. R.; Scheltema R. A. Efficient and robust proteome-wide approaches for cross-linking mass spectrometry. Nat. Protoc. 2018, 13, 2964–2990. 10.1038/s41596-018-0074-x. 30446747

[ref8] Kastritis P. L.; O’Reilly F. J.; Bock T.; Li Y.; Rogon M. Z.; Buczak K.; Romanov N.; Betts M. J.; Bui K. H.; Hagen W. J.; Hennrich M. L.; Mackmull M. T.; Rappsilber J.; Russell R. B.; Bork P.; Beck M.; Gavin A. C. Capturing protein communities by structural proteomics in a thermophilic eukaryote. Mol. Syst. Biol. 2017, 13, 936 10.15252/msb.20167412. 28743795 PMC5527848

[ref9] Schweppe D. K.; Chavez J. D.; Lee C. F.; Caudal A.; Kruse S. E.; Stuppard R.; Marcinek D. J.; Shadel G. S.; Tian R.; Bruce J. E. Mitochondrial protein interactome elucidated by chemical cross-linking mass spectrometry. Proc. Natl. Acad. Sci. U. S. A. 2017, 114, 1732–1737. 10.1073/pnas.1617220114. 28130547 PMC5321032

[ref10] Fasci D.; van Ingen H.; Scheltema R. A.; Heck A. J. R. Histone Interaction Landscapes Visualized by Crosslinking Mass Spectrometry in Intact Cell Nuclei. Mol. Cell. Proteomics 2018, 17, 2018–2033. 10.1074/mcp.RA118.000924. 30021884 PMC6166682

[ref11] Gotze M.; Iacobucci C.; Ihling C. H.; Sinz A. A Simple Cross-Linking/Mass Spectrometry Workflow for Studying System-wide Protein Interactions. Anal. Chem. 2019, 91, 10236–10244. 10.1021/acs.analchem.9b02372. 31283178

[ref12] Steigenberger B.; van den Toorn H. W. P.; Bijl E.; Greisch J. F.; Rather O.; Lubeck M.; Pieters R. J.; Heck A. J. R.; Scheltema R. A. Benefits of Collisional Cross Section Assisted Precursor Selection (caps-PASEF) for Cross-linking Mass Spectrometry. Mol. Cell. Proteomics 2020, 19, 1677–1687. 10.1074/mcp.RA120.002094. 32694122 PMC8015012

[ref13] Chavez J. D.; Lee C. F.; Caudal A.; Keller A.; Tian R.; Bruce J. E. Chemical Crosslinking Mass Spectrometry Analysis of Protein Conformations and Supercomplexes in Heart Tissue. Cell Syst. 2018, 6, 136–141.e5. 10.1016/j.cels.2017.10.017. 29199018 PMC5799023

[ref14] Beveridge R.; Stadlmann J.; Penninger J. M.; Mechtler K. A synthetic peptide library for benchmarking crosslinking-mass spectrometry search engines for proteins and protein complexes. Nat. Commun. 2020, 11, 742 10.1038/s41467-020-14608-2. 32029734 PMC7005041

[ref15] Erzberger J. P.; Stengel F.; Pellarin R.; Zhang S.; Schaefer T.; Aylett C. H. S.; Cimermancic P.; Boehringer D.; Sali A.; Aebersold R.; Ban N. Molecular Architecture of the 40SeIF1eIF3 Translation Initiation Complex. Cell 2014, 159, 1227–1228. 10.1016/j.cell.2014.11.001. 28898626 PMC5628955

[ref16] Fursch J.; Kammer K. M.; Kreft S. G.; Beck M.; Stengel F. Proteome-Wide Structural Probing of Low-Abundant Protein Interactions by Cross-Linking Mass Spectrometry. Anal. Chem. 2020, 92, 4016–4022. 10.1021/acs.analchem.9b05559. 32011863

[ref17] Leitner A.; Walzthoeni T.; Aebersold R. Lysine-specific chemical cross-linking of protein complexes and identification of cross-linking sites using LC-MS/MS and the xQuest/xProphet software pipeline. Nat. Protoc. 2014, 9, 120–137. 10.1038/nprot.2013.168. 24356771

[ref18] Gotze M.; Pettelkau J.; Fritzsche R.; Ihling C. H.; Schafer M.; Sinz A. Automated assignment of MS/MS cleavable cross-links in protein 3D-structure analysis. J. Am. Soc. Mass Spectrom. 2015, 26, 83–97. 10.1007/s13361-014-1001-1. 25261217

[ref19] Liu F.; Rijkers D. T. S.; Post H.; Heck A. J. R. Proteome-wide profiling of protein assemblies by cross-linking mass spectrometry. Nat. Methods 2015, 12, 1179–1184. 10.1038/nmeth.3603. 26414014

[ref20] Giese S. H.; Fischer L.; Rappsilber J. A Study into the Collision-induced Dissociation (CID) Behavior of Cross-Linked Peptides. Mol. Cell. Proteomics 2016, 15, 1094–1104. 10.1074/mcp.M115.049296. 26719564 PMC4813691

[ref21] Yang B.; Wu Y. J.; Zhu M.; Fan S. B.; Lin J.; Zhang K.; Li S.; Chi H.; Li Y. X.; Chen H. F.; Luo S. K.; Ding Y. H.; Wang L. H.; Hao Z.; Xiu L. Y.; Chen S.; Ye K.; He S. M.; Dong M. Q. Identification of cross-linked peptides from complex samples. Nat. Methods 2012, 9, 904–906. 10.1038/nmeth.2099. 22772728

[ref22] Chen Z. L.; Meng J. M.; Cao Y.; Yin J. L.; Fang R. Q.; Fan S. B.; Liu C.; Zeng W. F.; Ding Y. H.; Tan D.; Wu L.; Zhou W. J.; Chi H.; Sun R. X.; Dong M. Q.; He S. M. A high-speed search engine pLink 2 with systematic evaluation for proteome-scale identification of cross-linked peptides. Nat. Commun. 2019, 10, 3404 10.1038/s41467-019-11337-z. 31363125 PMC6667459

[ref23] Veit J.; Sachsenberg T.; Chernev A.; Aicheler F.; Urlaub H.; Kohlbacher O. LFQProfiler and RNP(xl): Open-Source Tools for Label-Free Quantification and Protein-RNA Cross-Linking Integrated into Proteome Discoverer. J. Proteome Res. 2016, 15, 3441–3448. 10.1021/acs.jproteome.6b00407. 27476824

[ref24] Eisele M. R.; Reed R. G.; Rudack T.; Schweitzer A.; Beck F.; Nagy I.; Pfeifer G.; Plitzko J. M.; Baumeister W.; Tomko R. J. Jr.; Sakata E. Expanded Coverage of the 26S Proteasome Conformational Landscape Reveals Mechanisms of Peptidase Gating. Cell Rep. 2018, 24, 1301–1315.e5. 10.1016/j.celrep.2018.07.004. 30067984 PMC6140342

[ref25] Sailer C.; Jansen J.; Erzberger J. P.; Stengel F. A comprehensive landscape of 60S ribosome biogenesis factors. Cell Rep. 2022, 38, 110353 10.1016/j.celrep.2022.110353. 35139378 PMC8884084

[ref26] Combe C. W.; Fischer L.; Rappsilber J. xiNET: cross-link network maps with residue resolution. Mol. Cell. Proteomics 2015, 14, 1137–1147. 10.1074/mcp.O114.042259. 25648531 PMC4390258

[ref27] Perez-Riverol Y.; Bai J.; Bandla C.; García-Seisdedos D.; Hewapathirana S.; Kamatchinathan S.; Kundu D. J.; Prakash A.; Frericks-Zipper A.; Eisenacher M.; Walzer M.; Wang S.; Brazma A.; Vizcaíno J. A. The PRIDE database resources in 2022: a hub for mass spectrometry-based proteomics evidences. Nucleic Acids Res. 2022, 50, D543–D552. 10.1093/nar/gkab1038. 34723319 PMC8728295

[ref28] Fischer L.; Rappsilber J. Quirks of Error Estimation in Cross-Linking/Mass Spectrometry. Anal. Chem. 2017, 89, 3829–3833. 10.1021/acs.analchem.6b03745. 28267312 PMC5423704

[ref29] Walzthoeni T.; Claassen M.; Leitner A.; Herzog F.; Bohn S.; Forster F.; Beck M.; Aebersold R. False discovery rate estimation for cross-linked peptides identified by mass spectrometry. Nat. Methods 2012, 9, 901–903. 10.1038/nmeth.2103. 22772729

[ref30] Lenz S.; Sinn L. R.; O’Reilly F. J.; Fischer L.; Wegner F.; Rappsilber J. Reliable identification of protein-protein interactions by crosslinking mass spectrometry. Nat. Commun. 2021, 12, 3564 10.1038/s41467-021-23666-z. 34117231 PMC8196013

[ref31] Rinner O.; Seebacher J.; Walzthoeni T.; Mueller L. N.; Beck M.; Schmidt A.; Mueller M.; Aebersold R. Identification of cross-linked peptides from large sequence databases. Nat. Methods 2008, 5, 315–318. 10.1038/nmeth.1192. 18327264 PMC2719781

