# Supplementary material for: Mono- and Intralink Filter (Mi-Filter) To Reduce False Identifications in Cross-Linking Mass Spectrometry Data
Source: Anal Chem. 2022 Dec 12;94(51):17751–6. doi: 10.1021/acs.analchem.2c00494 (PMC9798375; doi:10.1021/acs.analchem.2c00494)
Supplement: Supplementary file 1 — ac2c00494_si_001.pdf [file ac2c00494_si_001.pdf]

Supporting Information

A mono- and intralink filter (mi-filter) to reduce false identifications in cross-linking mass spectrometry data

Xingyu Chen[1,2,#], Carolin Sailer[1,2,#], Kai Michael Kammer[1,2], Julius Fürsch[1,2], Markus R. Eisele[3], Eri Sakata [3,4], Riccardo Pellarin[5] & Florian Stengel[1,2]

1 University of Konstanz, Department of Biology, Universitätsstrasse 10, 78457 Konstanz, Germany

2 Konstanz Research School Chemical Biology, University of Konstanz, Universitätsstrasse 10, 78457 Konstanz, Germany

3 Department of Molecular Structural Biology, Max Planck Institute of Biochemistry, 82152 Martinsried, Germany

4 Institute for Auditory Neuroscience, University Medical Center Göttingen, 37077 Göttingen, Germany

5 Structural Bioinformatics Unit, Department of Structural Biology and Chemistry, Institut Pasteur, CNRS UMR 3528, 28 rue du Docteur Roux, 75015 Paris, France

Email address: florian.stengel@uni-konstanz.de

Table of contents

Supplementary Figure 1. Relative abundance of inter-protein cross-links with and without additional mono- or intra-protein cross-link.

Supplementary Figure 2. Mapped distances of all inter-protein cross-links within the mi-filtered 26S proteasome dataset at ld 25.

Supplementary Table 1. Mapping of identified cross-links with and without mi-filtering onto a high-resolution structure for the proteasome dataset.

Supplementary Data 1. Containing Supplemental Tables 1 to 5 (xlsx), showing identified crosslinks of the 26S proteasome from *S. cerevisiae* crosslinked with and without mi-filter. Dataset referring to Figure 3A, 3B, 4 and Figure S2.

Supplementary Data 2. Containing Supplemental Tables 1 and 2 (xlsx), showing identified crosslinks from 60S ribosome biogenesis intermediates in S. cerevisiae crosslinked with and without mi-filter. Dataset referring to Figure 3C, 3D and Figure S1.

Supplementary Data 3. Containing Supplemental Tables 1 to 2 (xlsx), showing identified crosslinks of *S. cerevisiae* lysate crosslinked with and without mi-filter.
Dataset referring to Figure 3E, 3F.

Supplementary Figures

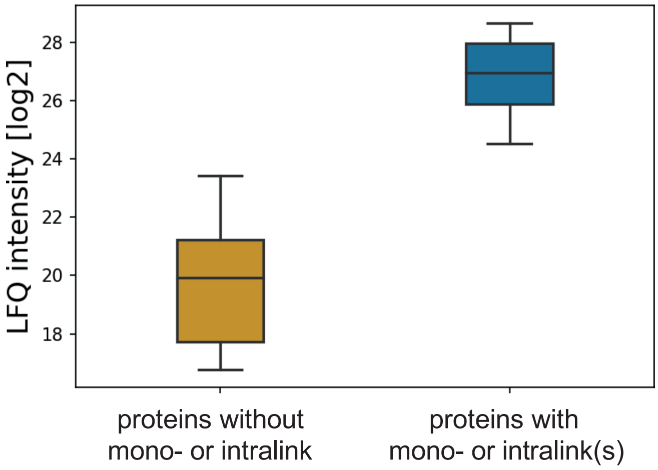

Supplementary Figure 1: Relative abundance of proteins without mono- or intra-protein link that were filtered-out by the *mi-filter* (yellow) compared to the relative abundance of proteins with a mono- or intra-protein link (blue). LFQ intensities were calculated based on peptides which were not modified by the crosslinking reagent DSS.

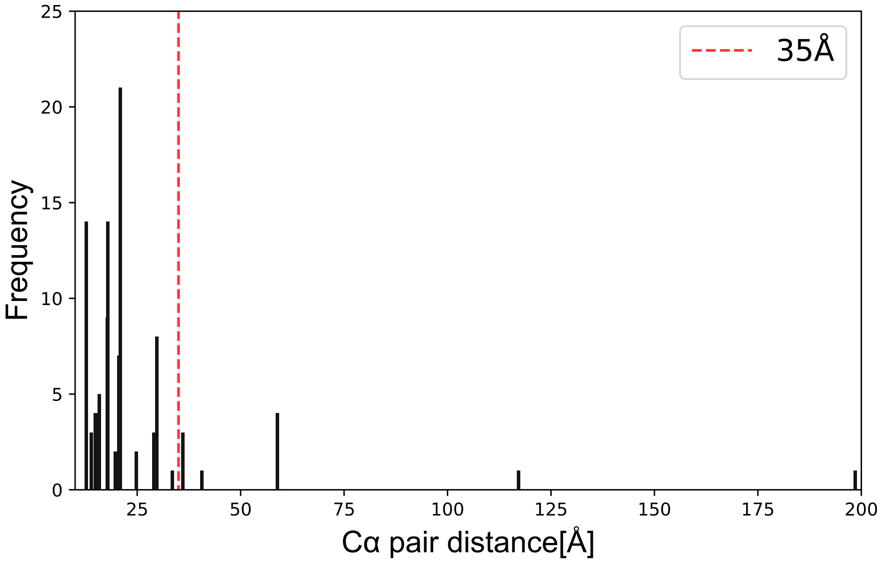

Supplementary Figure 2: Histogram of mapped distances of all inter-protein cross-links at ld 25 within the mi-filtered 26S proteasome data ("proteome-wide setting" containing the 34 proteins of the 26S proteasome plus the 200 most abundant proteins in *S. cerevisiae* as annotated in the PAX database (https://pax-db.org/)) versus their frequency. The 35A° threshold (i.e. the maximal lysine Cα-Cα distance that our crosslinker can bridge) is indicated as a red dotted line. In total 114 inter-protein cross-links could be mapped the high-resolution structure of the S. cerevisiae 26S proteasome (PDB 4CR2) of which 104 were below 35A° (138 and 115 before application of the mi-filter, respectively).

Supplementary Table

sample_name	links(<= 35Å)	links(all)	links(>35Å)	percentage	sensitivity
proteasome ld20	204	348	144	0,59	
mi-filtered ld20	188	288	100	0,65	0,92
proteasome ld25	164	211	47	0,78	
mi-filtered ld25	151	178	27	0,85	0,92
proteasome-ld28	128	157	29	0,82	
mi-filtered ld28	120	136	16	0,88	0,94
proteasome ld32	71	88	17	0,81	
mi-filtered ld32	65	72	7	0,90	0,92

Supplementary Table 1: Mapping of identified inter-protein cross-links for the proteasome dataset from Figure 3A onto PDB 4CR2 with and without mi-filtering demonstrates good sensitivity as the majority (> 90%) of bona-fide true positive links (i.e. links below 35 A° Cα-Cα distance) for the various cut-offs are retained after application of the mi-filter. Discrepancies between all links identified and mapped links are due to missing parts within the solved high-resolution PDB.
